# Lumpy Skin Disease Virus Infection in Free-Ranging Indian Gazelles (*Gazella bennettii*), Rajasthan, India

**DOI:** 10.3201/eid2907.230043

**Published:** 2023-07

**Authors:** Shashi Bhushan Sudhakar, Niranjan Mishra, Semmannan Kalaiyarasu, Khusboo Ahirwar, Suchismita Chatterji, Omprakash Parihar, Vijendra Pal Singh, Aniket Sanyal

**Affiliations:** ICAR-National Institute of High Security Animal Diseases, Bhopal, India (S.B. Sudhakar, N. Mishra, S. Kalaiyarasu, K. Ahirwar, V.P. Singh, A. Sanyal);; Regional Disease Diagnostic Centre, Bikaner, India (S. Chatterji, O. Parihar)

**Keywords:** lumpy skin disease virus, viruses, wildlife, Indian gazelle, Gazella bennettii, genetic analysis, India

## Abstract

Near a zoo in Bikaner, India, 2 free-ranging Indian gazelles (*Gazella bennettii*) displayed nodular skin lesions. Molecular testing revealed lumpy skin disease virus (LSDV) infection. Subsequent genome analyses revealed LSDV wild-type strain of Middle Eastern lineage. Evidence of natural LSDV infection in wild gazelles in this area indicates a broadening host range.

Lumpy skin disease (LSD), caused by lumpy skin disease virus (LSDV) of the genus *Capripoxvirus*, is a notifiable transboundary disease of domestic cattle and has recently spread from eastern Europe and Russia to South, East, and Southeast Asia (*1*). Although cattle are the principal hosts, natural LSDV infection has been reported sporadically in wildlife in Africa and Asia (*2*–*5*).

The Indian gazelle (*Gazella bennettii*), a free-ranging ungulate (family *Bovidae*, subfamily *Antilopinae*), is native to the arid regions of India, Pakistan, Iran, and Afghanistan; most live in the Rajasthan state of India (*6*). Recently, lethal LSDV infection was reported in a captive giraffe (*Giraffa camelopardalis*) in Vietnam (*5*), and clinical disease was reported in wildlife in Thailand (*7*). However, the epidemiologic role of wildlife has not been elucidated, and LSDV infection previously has not been detected in Indian gazelles. In addition, information on clinical disease and genetic profile of LSDV from wildlife is scarce. We report detection and genetic characterization of LSDV from wild Indian gazelles in Rajasthan, India.

## The Study

**Figure 1:**
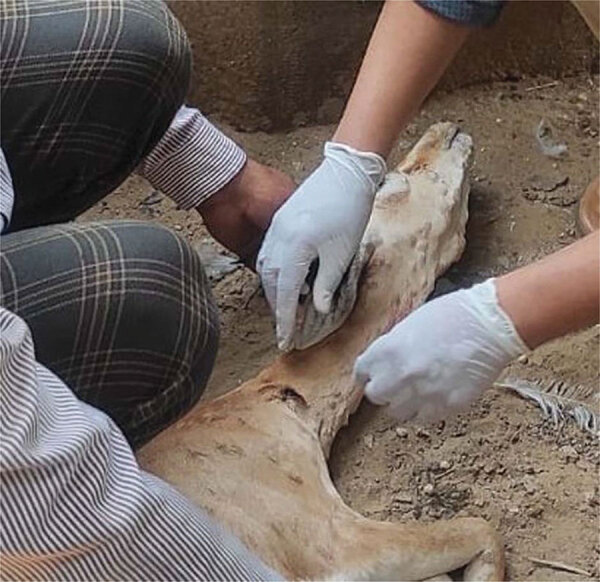
Clinical observations of lumpy skin disease virus infection in free-ranging Indian gazelles (*Gazella bennettii*), Rajasthan, India. Photograph shows a female Indian gazelle with multiple circumscribed skin nodules of varying sizes over the entire body, including the face and neck region.

In August 2022, two free-ranging female Indian gazelles with skin lesions resembling LSD were rescued and quarantined for veterinary care at a zoo in Bikaner, Rajasthan, India. The animals had high fever, vesicles in the mouth, nasal discharge, ocular and oral discharge, and generalized skin nodules all over the body, including the neck and face (Figure 1). Skin and whole blood samples from the affected animals were investigated by PCR and real-time PCR. Both the animals died under veterinary care after 3 days but could not be necropsied for histology and further analysis.

We performed various real-time PCRs and PCRs using DNA extracted from skin lesions and blood by using a capripoxvirus-screening real-time PCR (*8*). Results for skin samples were LSDV-positive but for blood samples were LSDV-negative (Appendix Figure 1, panel A). We performed real-time PCR specific for LSDV wild-type strain (*9*), which showed positive results for skin samples, confirming natural LSDV infection (Appendix Figure 1, panel B). PCR of skin and blood samples were negative for bovine herpes virus type 2, buffalopox virus, cowpox virus, pseudo-cowpox virus, and bovine papular stomatitis virus (*10*). Although the exact cause of death of the 2 Indian gazelles could not be ascertained, LSDV-associated death is likely because the animals tested negative for other related cattle viral pathogens.

To determine the genetic profile of the LSDV strains, we conducted PCR amplification for 3 complete LSDV genes, the LSDV011 G-protein-coupled-chemokine-like receptor (GPCR), LSDV036 RNA polymerase 30-kDa polypeptide (RPO30), and LSDV126 extracellular enveloped virus (EEV), as described in our previous study (*11*). We also analyzed skin tissues of 2 LSDV-positive domestic cattle from the nearby area in Rajasthan for comparison. We determined the GPCR, RPO30, and EEV full gene sequences by Sanger sequencing and deposited the sequences in GenBank (accession nos. OP893954–65). We performed phylogenetic analysis by using MEGA version 7.0 (*12*). We found that LSDV sequences from both the gazelles and 2 domestic cattle were identical and subjected 1 sequence from each animal to further genetic analysis.

The GPCR nucleotide sequence alignment showed that the LSDV strains from the Indian gazelles and local cattle had a 12-nt deletion, as previously seen in LSDV wild-type strains of the SG-1 lineage from the Middle East, Europe, and the Balkans (Table**)**. In contrast, all LSDVs reported in India since 2019 had a 12-nt insertion, as observed in ancestral wild-type strains of SG-2 lineage from Kenya. Those results suggest the emergence of LSDV SG-1 lineage in India. In addition, the phylogenetic tree analysis of GPCR showed that LSDV from the Indian gazelles clustered with the LSDV wild-type strains (Appendix Figure 2).

**Table:** Results of GPCR nucleotide sequence alignment of lumpy skin disease virus infection in free-ranging Indian gazelles (*Gazella bennettii*), Rajasthan, India*

Sample no.	Virus strain	GenBank accession no.	12-nt deletion of in GPCR (96–107)
1	Indian Gazelle/IND/LSDV/BKN-6/2022	OP893960	Y
2	Cattle/IND/LSDV/BKN-2	OP893958	Y
3	Cattle/IND/LSDV/L06/2022	SRX17592135	N
4	Cattle/IND/LSDV/L09/2022	SRX17592137	N
5	Cattle/LSDV/IND/ODI/5RK_LT/2019	MW452639	N
6	Cattle/LSDV/IND/ODI/77BP-LT/2019	MW452646	N
7	Cattle/LSDV/IND/WB/JS9_LT/2019	MW452649	N
8	Cattle/LSDV/Bangladesh/Dhaka/2019	MT448698	N
9	Cattle/LSDV/Kenya/KSGP-O240/Kenyavac	KJ818281	N
10	Cattle/LSDV/Turkey/Pendik/2014	MN995838	Y
11	Cattle/LSDV/Serbia/Bujanovac/2016	KY702007	Y
12	Cattle/LSDV/Kazakhstan/Kubash/2016	MN642592	Y
13	Cattle/LSDV/Greece/Evros/2015	KY829023	Y
14	Cattle/ South Africa/OBP vaccine	KX764645	N
15	Cattle/South Africa/Herbivac vaccine	KX764644	N
16	Cattle/LSDV/South Africa/SIS-Lumpyvax Vaccine	KX764643	N
17	Cattle/LSDV/Russia/Saratov/2017	MH646674	N
18	Cattle/LSDV/Russia/Udmurthiya/2019	MT134042	N
19	Cattle/LSDV/ Kenya/1958	MNO72619	N
20	Cattle/LSDV/Russia/Dagestan/2015	MH893760	Y
21	Springbok/LSDV/South Africa/RSA/06/2011	FJ869374	Y
22	Cattle/LSDV/Kenya/Neethling Warmaths LW	AF409137	Y
23	Cattle/LSDV/Egypt/AHRI21/2019	MN271744	Y
24	Cattle/LSDV/Kenya/Neethling/2490	AF325528	N
25	Cattle/LSDV/China/Xinjiang/2019	MN598006	N
26	Cattle/LSDV/Kenya/KS-1	KJ818283	N
27	Giraffe/LSDV/Vietnam/2021	MZ966326	N
28	SPPVaccine/India/Ranipet vaccine	KF495236	N
29	SPPV/Turkey/Vaccine	MN072631	N
30	GTPV/India/ Uttarkashi/Vaccine	KF495242	N
31	GTPV/Iran/Gorgan/Vaccine	KX576657	N

*Samples 1 and 2 were those from this study. Multiple sequence alignment was conducted by using MEGA version 7.0 (*12*). GTPV, goatpox virus; GPCR, G-protein-coupled-chemokine-like receptor (LSDV011); SPPV, sheeppox virus.

**Figure 2:**
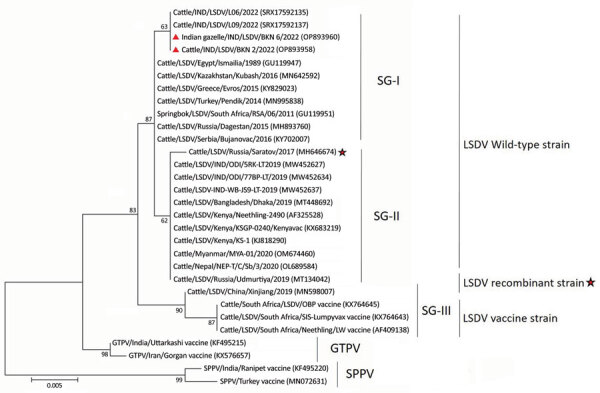
Phylogenetic tree of LSDV from infected free-ranging Indian gazelles (*Gazella bennettii*), Rajasthan, India, compared with reference strains from GenBank. LSDV tree is based on complete LSDV036 RNA polymerase 30-kDa polypeptide (RPO30) gene sequences using maximum-likelihood analysis combined with 1,000 bootstrap replicates. LSDV from Indian gazelle clustered with LSDV wild-type strains from Africa, the Middle East, and Europe. Triangles indicate sequences obtained from this study; stars indicate sequences of LSDV recombinant strains from GenBank. Scale bar indicates nucleotide substitutions per site. GenBank accession numbers are provided. GTPV, goatpox virus; LSDV, lumpy skin disease virus; SPPV, sheeppox virus.

Further phylogenetic analysis of the complete RPO30 gene, commonly used for LSDV genetic tree calculations, showed that LSDV from the gazelles and local cattle in a 2022 LSD outbreak clustered with the LSDV wild-type strains of SG-1 lineage, but they diverged from the main branch in a separate cluster (Figure 2). Those data confirmed emergence of LSDV variants of SG-1 lineage in India, indicating a change in the genetic makeup of recent LSDV wild-type strains. This finding also was supported by our results of EEV nucleotide sequence alignment, which showed that LSDVs from the gazelles had 1 unique mutation (G253A) and 2 mutations (G178A and A459G) that are similar to other wild-type strains of LSDV SG-1 lineage (Appendix Figure 3). In addition, the EEV phylogenetic analysis showed that LSDV from the gazelles clustered with LSDV SG-1 lineage (Appendix Figure 4).

## Conclusions

We detected LSDV in 2 diseased free-ranging Indian gazelles in Rajasthan, India. The 2 gazelles eventually died. The clinical manifestations of their disease were akin to those for LSD in domestic cattle. The findings demonstrated emergence of LSD in wildlife in India and susceptibility of the wild *G. bennetti* species to natural LSDV infection. To our knowledge, LSDV-associated death has not been reported in free-ranging wildlife, and most LSDV infections in wildlife are asymptomatic, despite sporadic reports of clinical disease (*5*–*7*) and a single report of death in a captive giraffe (*5*). However, further investigations are needed to assess effects of LSDV infection in the Indian gazelle population and other susceptible wildlife.

Genetic and phylogenetic analysis of LSDV GPCR, RPO30, and EEV sequences revealed that the LSDV from the Indian gazelles clustered with the LSDV wild-type strains of SG-1 lineage commonly circulating in the Middle East, the Balkans, and Europe (*13*). In contrast, since its emergence in India in 2019, all the LSDV strains circulating in domestic cattle have belonged to the ancestral LSDV wild-type strains of SG-2 lineage from Kenya (*10*,*11*). Hence, our findings suggest a new introduction of LSDV of exotic origin into India.

In conclusion, we found LSDVs in Indian gazelles and local domestic cattle that were phylogenetically similar, reinforcing the hypothesis that susceptible wildlife can become infected with LSDV circulating in cattle in the region, as reported in previous studies (*5*,*14*,*15*). Our findings demonstrate that the host range of LSDV is expanding and free-ranging wildlife in Asia is susceptible to LSDV. Minimizing contacts between wildlife and cattle during LSD outbreaks might help limit cross-species transmission. Continued monitoring is needed to assess the impact of LSDV on gazelles and other wild and domestic ruminants in India.

Appendix Additional information on lumpy skin disease virus infection in free-ranging Indian gazelles (*Gazella bennettii*), Rajasthan, India.
