# Supplementary material for: Lumpy Skin Disease Virus Infection in Free-Ranging Indian Gazelles (Gazella bennettii), Rajasthan, India
Source: Emerg Infect Dis. 2023 Jul;29(7):1407–10. doi: 10.3201/eid2907.230043 (PMC10310394; doi:10.3201/eid2907.230043)
Supplement: Appendix — Additional information on lumpy skin disease virus infection in free-ranging Indian gazelles (Gazella bennettii), Rajasthan, India. [file 23-0043-Techapp-s1.pdf]

Article DOI: https://doi.org/10.3201/eid2907.230043

EID cannot ensure accessibility for supplementary materials supplied by authors. Readers who have difficulty accessing supplementary content should contact the authors for assistance.

Lumpy Skin Disease Virus Infection in Free-Ranging Indian Gazelles (*Gazella bennettii*), Rajasthan, India

Appendix

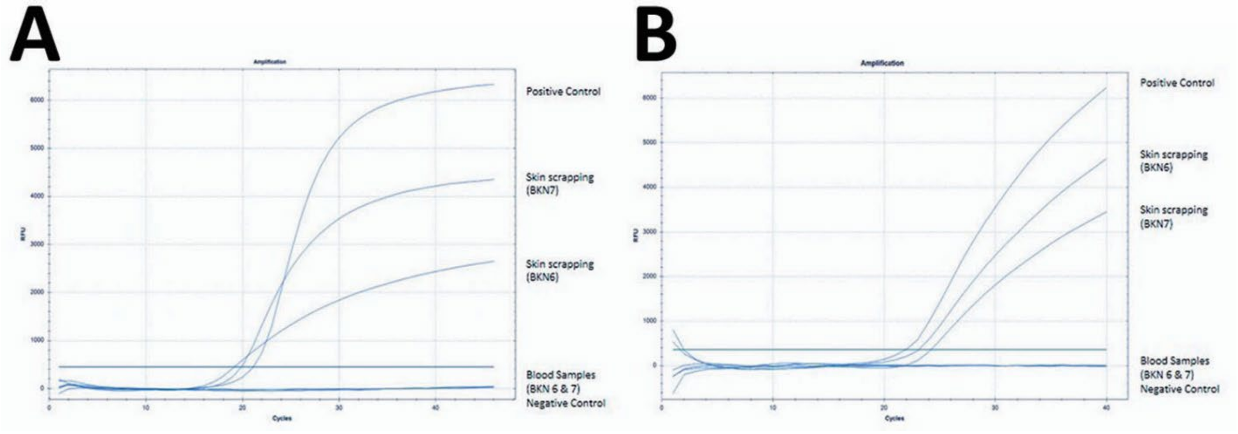

Appendix Figure 1. Detection of LSDV genome in Indian gazelle by qPCR. A) Test results of skin scrapping and blood samples from Indian gazelles (BKN6, BKN7) with pan-capripoxvirus generic real-time PCR (*1*). B) Test results of skin scrapping and blood samples from Indian gazelles (BKN6, BKN7) with LSDV wild-type specific real-time PCR (*2*). LSDV, lumpy skin virus disease.

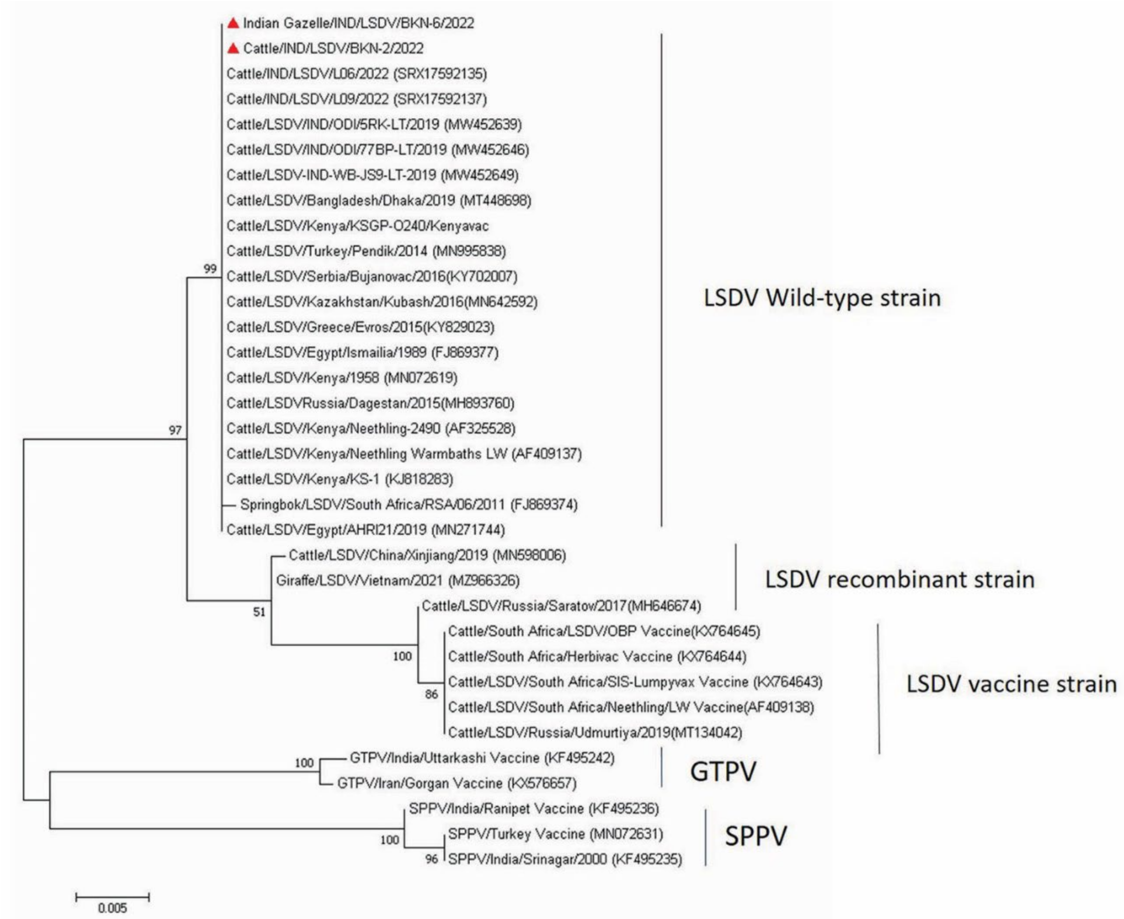

Appendix Figure 2. Maximum likelihood (ML) phylogenetic tree based on complete LSDV GPCR gene sequences from Indian gazelles (*Gazella bennettii*). We performed multiple alignment and phylogenetic analysis using MEGA version 7.0 (*3*) showing genetic relationship between the LSDV strains obtained in this study and other selected LSDV, GTPV, and SPPV strains from GenBank. Red triangles indicate sequences obtained in this study. Scale bar indicates nucleotide substitutions per site. GTPV, goatpox virus; LSDV, lumpy skin disease virus; SPPV, sheeppox virus.

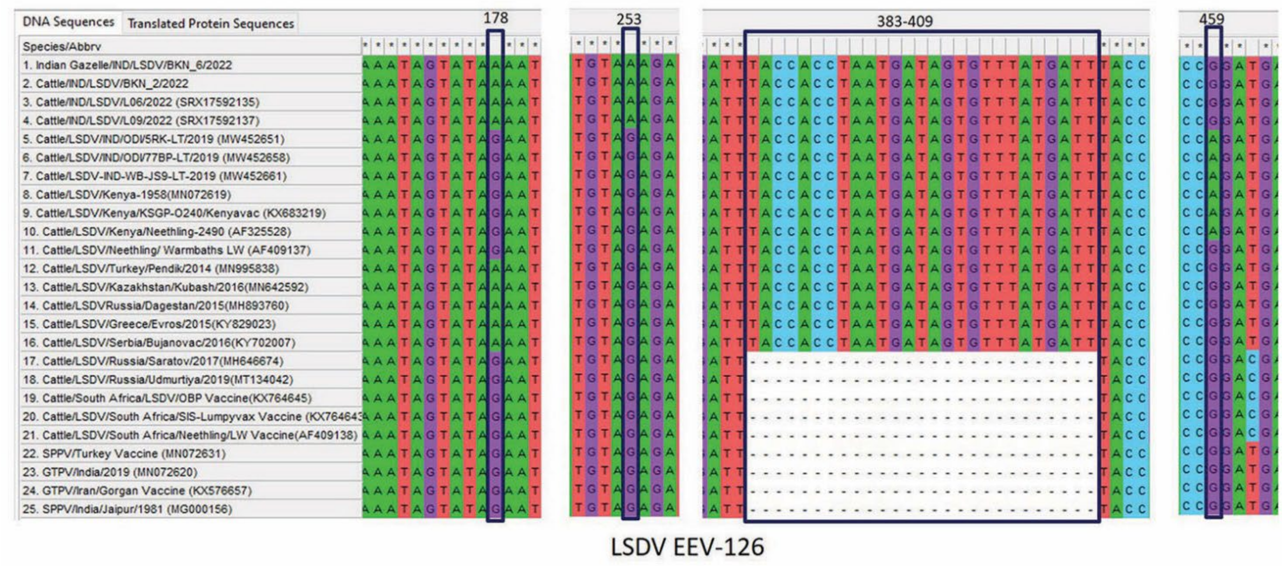

Appendix Figure 3. Multiple alignment of complete EEV gene nucleotide sequences of LSDV from Indian gazelle (*Gazella bennettii*) and local cattle. Sequences from this study were compared with representative LSDV, GTPV, and SPPV sequences from GenBank. Identical nucleotides are shown in same color and differences are shown in different color shades. Important nucleotide differences found in LSDV from Indian gazelle are highlighted in a box. Hyphens correspond to absence of nucleotide at corresponding positions and blank shaded area denotes non-availability of sequences for comparison. LSDV from Indian gazelle showed absence of a 27-nt deletion (position 383–409), a signature found only in LSDV wild-type strains, which is highlighted with a rectangle. GTPV, goatpox virus; LSDV, lumpy skin disease virus; SPPV, sheeppox virus.

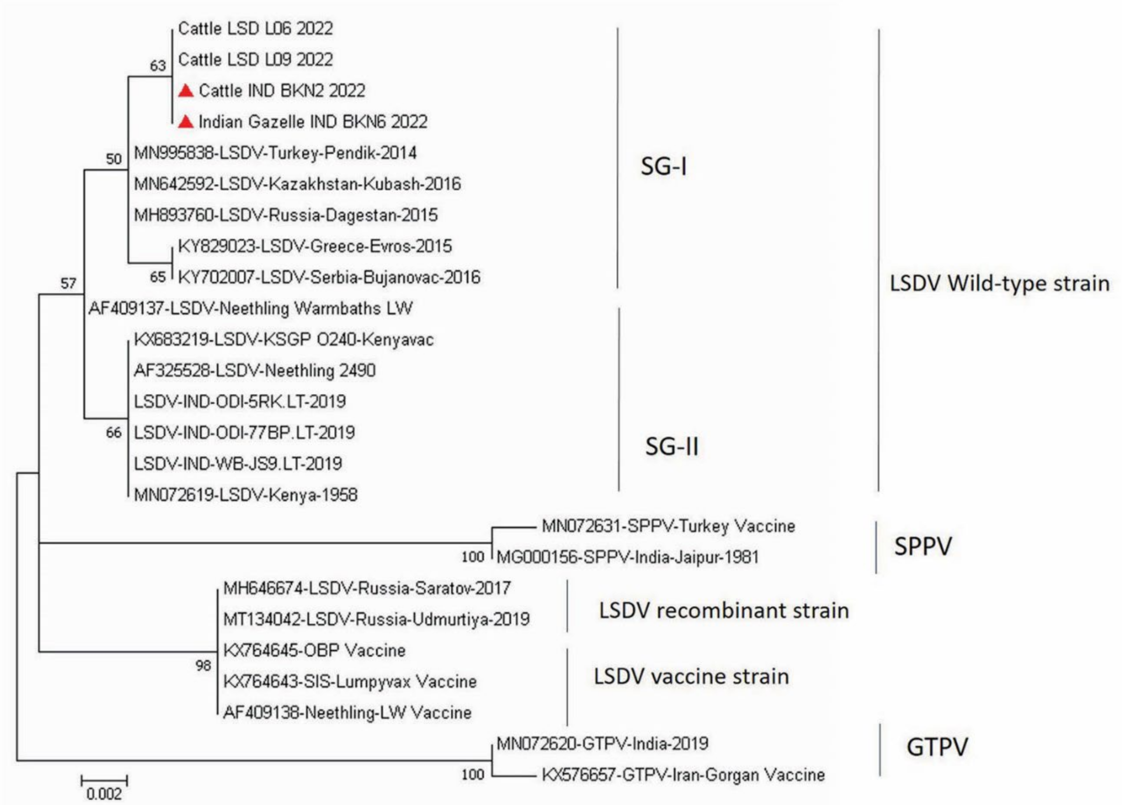

Appendix Figure 4. Maximum likelihood (ML) phylogenetic tree based on complete EEV gene sequences. The genetic relationship between the LSDV strains obtained in this study and other selected LSDV, GTPV, and SPPV strains was determined by multiple sequence alignment and phylogenetic analysis using MEGA version 7.0 (*3*). Red triangles indicate sequences obtained in this study. Scale bar indicates nucleotide substitutions per site. GTPV, goatpox virus; LSDV, lumpy skin disease virus; SPPV, sheeppox virus.

References

1. Bowden TR, Babiuk SL, Parkyn GR, Copps JS, Boyle DB. Capripoxvirus tissue tropism and shedding: a quantitative study in experimentally infected sheep and goats. Virology. 2008;371:380–93. PubMed https://doi.org/10.1016/j.virol.2007.10.002

2. Pestova YE, Artyukhova EE, Kostrova EE, Shumoliva IN, Kononov AV, Sprygin AV. Real time PCR for the detection of field isolates of lumpy skin disease virus in clinical samples from cattle. Agric Biol. 2018;53:422–9. https://doi.org/10.15389 /agrobiology.2018.2.422eng

3. Kumar S, Stecher G, Tamura K. MEGA7: Molecular evolutionary genetics analysis version 7.0 for bigger datasets. Mol Biol Evol. 2016;33:1870–4. PubMed https://doi.org/10.1093/molbev/msw054
